# Supplementary material for: High‐throughput profiling and analysis of plant responses over time to abiotic stress
Source: Plant Direct. 2017 Oct 25;1(4):e00023. doi: 10.1002/pld3.23 (PMC6508565; doi:10.1002/pld3.23)
Supplement: Supplementary file 1 [file PLD3-1-e00023-s001.pdf]

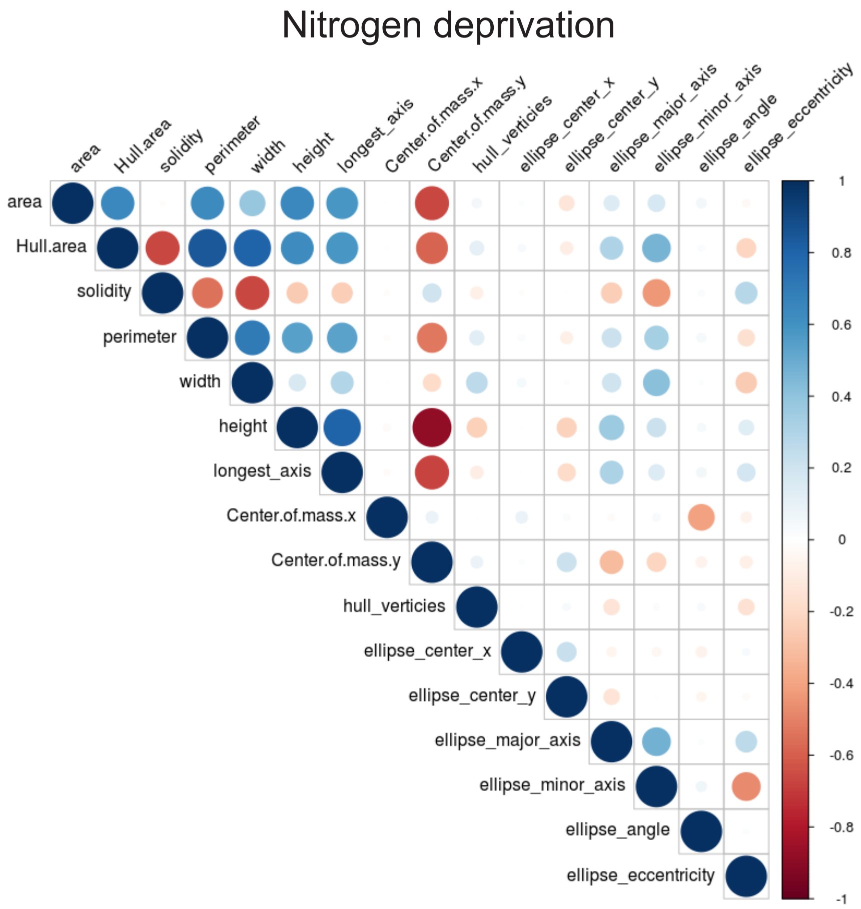

Figure S1. All shape parameterizations returned from PlantCV had correlations calculated to all other shapes. Correlation is on a scale from -1 to 1 indicating inversely or directly correlated and is being shown in color from red to blue. Radius of the circle in each cell is on a scale between 0 and 1 which corresponds to the absolute value of the correlation.
