# Supplementary material for: High‐throughput profiling and analysis of plant responses over time to abiotic stress
Source: Plant Direct. 2017 Oct 25;1(4):e00023. doi: 10.1002/pld3.23 (PMC6508565; doi:10.1002/pld3.23)
Supplement: Supplementary file 2 [file PLD3-1-e00023-s002.pdf]

Genotype

Source	Chisq	DF	p-value
Nitrogen	415.9	2	<0.0001
Genotype	130.1	29	<0.0001
Nitrogen x Genotype	79.27	58	0.033
Nitrogen x Time	10516	2	<0.0001
Genotype x Time	197.4	29	<0.0001
Nitrogen x Genotype x Time	91.93	58	**0.003**

Type

Source	Chisq	DF	p-value
Nitrogen	1875	2	<0.0001
Type	12.86	2	0.002
Nitrogen x Type	1.844	4	0.764
Nitrogen x Time	4085	2	<0.0001
Type x Time	16.51	2	0.001
Nitrogen x Type x Time	2.321	4	0.677

Photoperiod

Source	Chisq	DF	p-value
Nitrogen	3512	2	<0.0001
Photoperiod	9.017	1	0.003
Nitrogen x Photoperiod	9.147	2	0.01
Nitrogen x Time	7963	2	<0.0001
Photoperiod x Time	1.499	1	0.221
Nitrogen x Photoperiod x Time	7.585	2	**0.023**

Race

Source	Chisq	DF	p-value
Nitrogen	2298	2	<0.0001
Race	26.6	9	0.002
Nitrogen x Race	20.06	18	0.329
Nitrogen x Time	5338	2	<0.0001
Race x Time	39.95	9	<0.0001
Nitrogen x Race x Time	17.55	18	0.486

Figure S2. Tables showing results of ANOVA indicating significance of experimental variation explained by either genotype, type, photoperiod or race as found by Wald's Chi-Square tests with their associated degrees of freedom (DF). Significant p-value < 0.1, bold. All three nitrogen treatments are included in the calculations.
