# Supplementary material for: High‐throughput profiling and analysis of plant responses over time to abiotic stress
Source: Plant Direct. 2017 Oct 25;1(4):e00023. doi: 10.1002/pld3.23 (PMC6508565; doi:10.1002/pld3.23)
Supplement: Supplementary file 3 [file PLD3-1-e00023-s003.pdf]

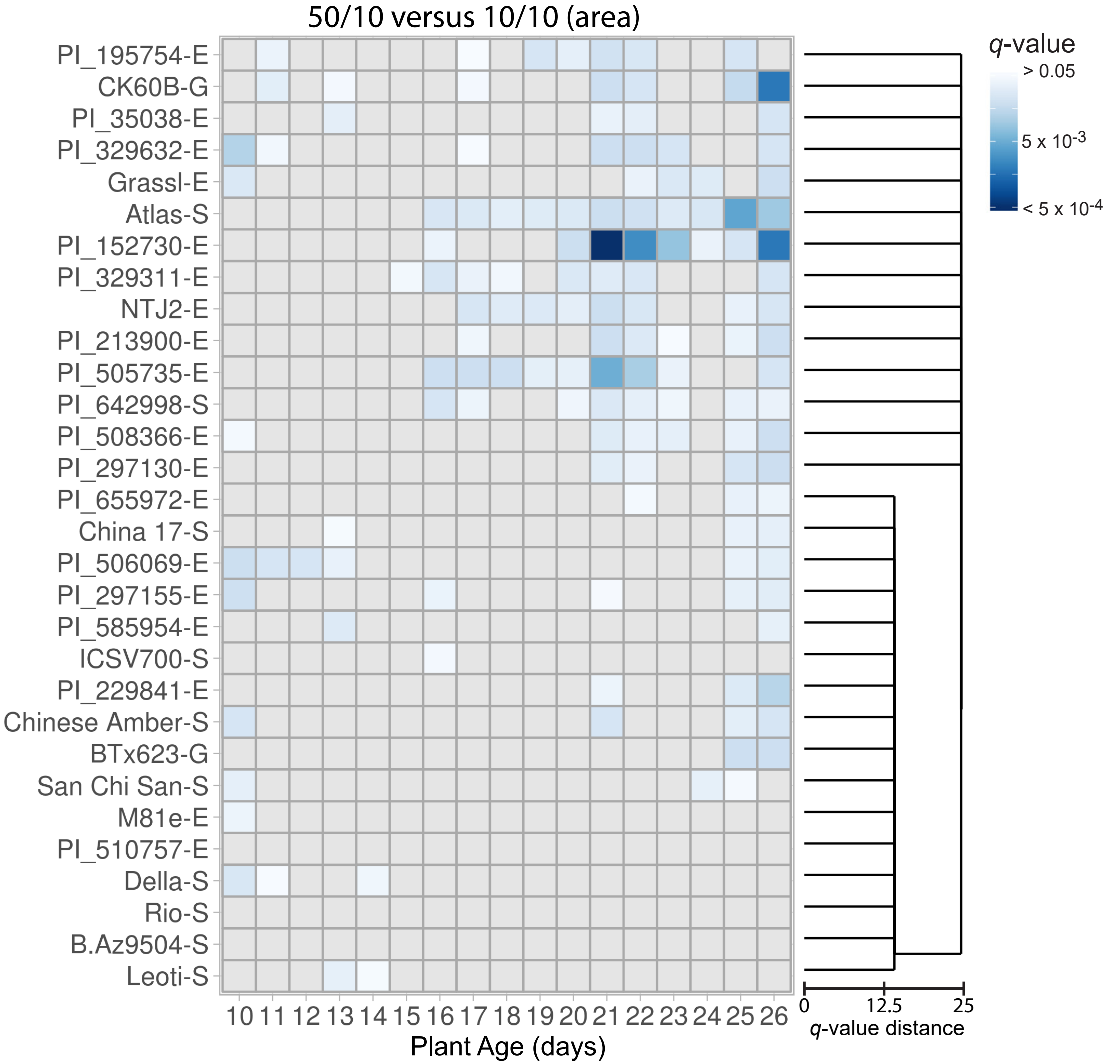

Figure S3. Statistical analysis of differences between 50/10 and 10/10 groups from the nitrogen deprivation experiment in area over time (bottom, plant age) for the 30 sorghum genotypes analyzed. q-values for the heat map are indicated in blue, with darkest coloring representing most significance. The Canberra distance-based cluster dendrogram (right) was generated from calculated q-values.
