# Supplementary material for: High‐throughput profiling and analysis of plant responses over time to abiotic stress
Source: Plant Direct. 2017 Oct 25;1(4):e00023. doi: 10.1002/pld3.23 (PMC6508565; doi:10.1002/pld3.23)
Supplement: Supplementary file 4 [file PLD3-1-e00023-s004.pdf]

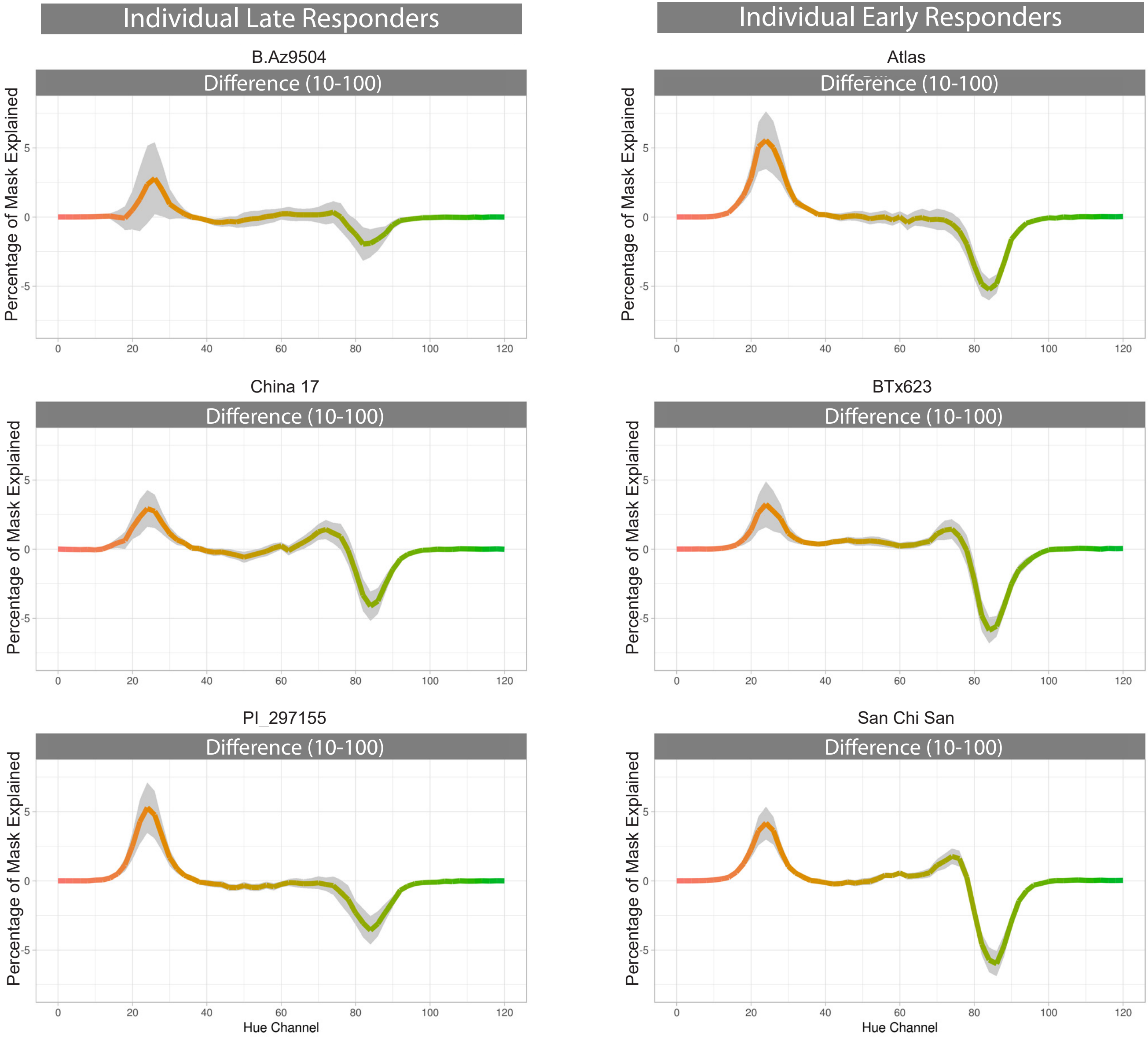

Figure S4. Color changes in individual late and early responding genotypes when the peak experimental effects were observed (day 13). To make the figure average histograms from the indicated genotypes within the 100% and 10% treatment groups were subtracted from one another. Grey areas indicate standard error.
