# Supplementary material for: High‐throughput profiling and analysis of plant responses over time to abiotic stress
Source: Plant Direct. 2017 Oct 25;1(4):e00023. doi: 10.1002/pld3.23 (PMC6508565; doi:10.1002/pld3.23)
Supplement: Supplementary file 5 [file PLD3-1-e00023-s005.pdf]

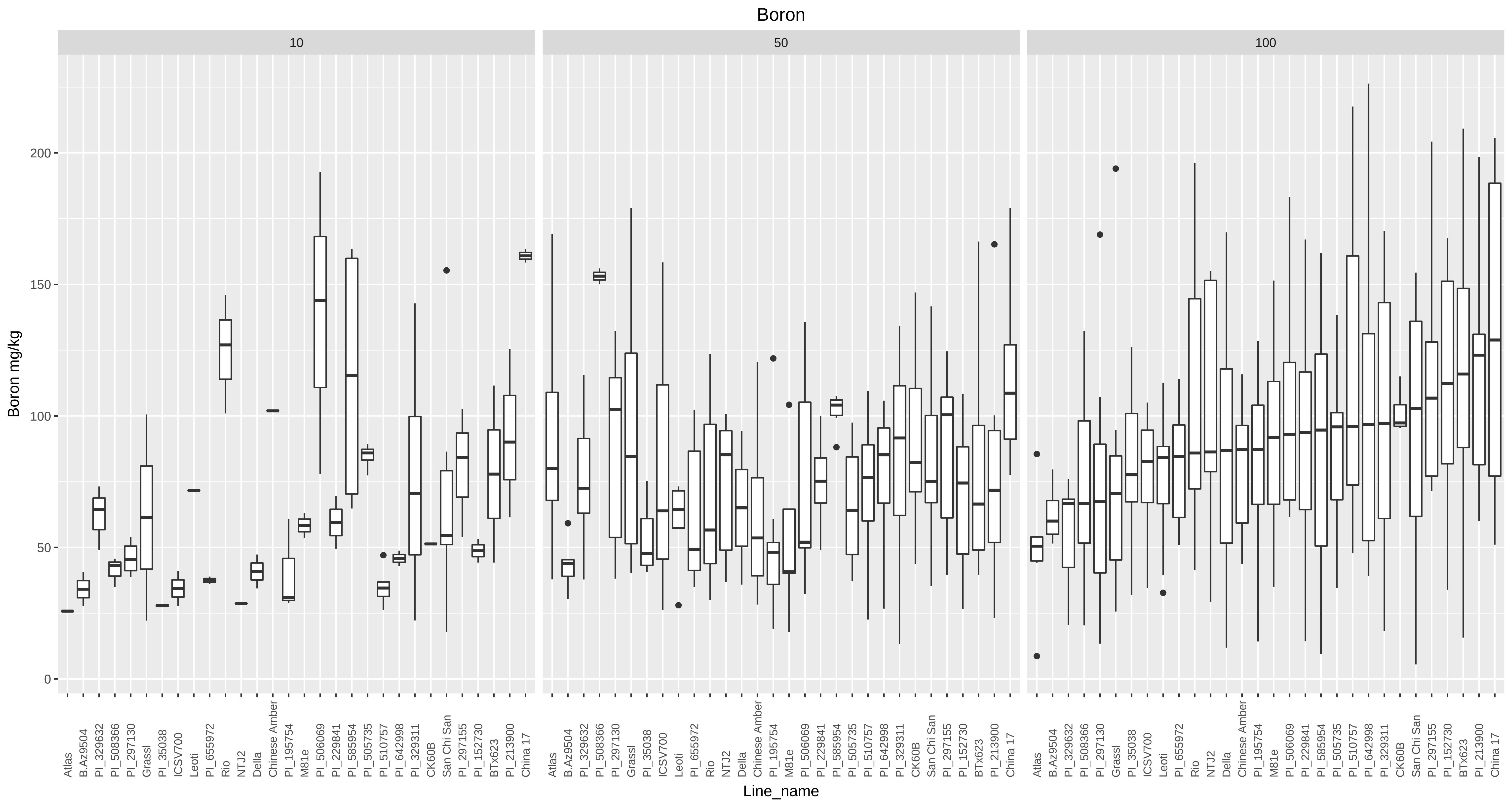
Boron
10
50
100
Boron mg/kg
0
50
100
150
200
Line_name
Atlas
B.Az9504
PI_329632
PI_508366
PI_297130
Grassl
PI_35038
ICSV700
Leoti
PI_655972
Rio
NTJ2
Della
Chinese Amber
PI_195754
M81e
PI_506069
PI_229841
PI_585954
PI_505735
PI_510757
PI_642998
PI_329311
CK60B
San Chi San
PI_297155
PI_152730
BTx623
PI_213900
China 17

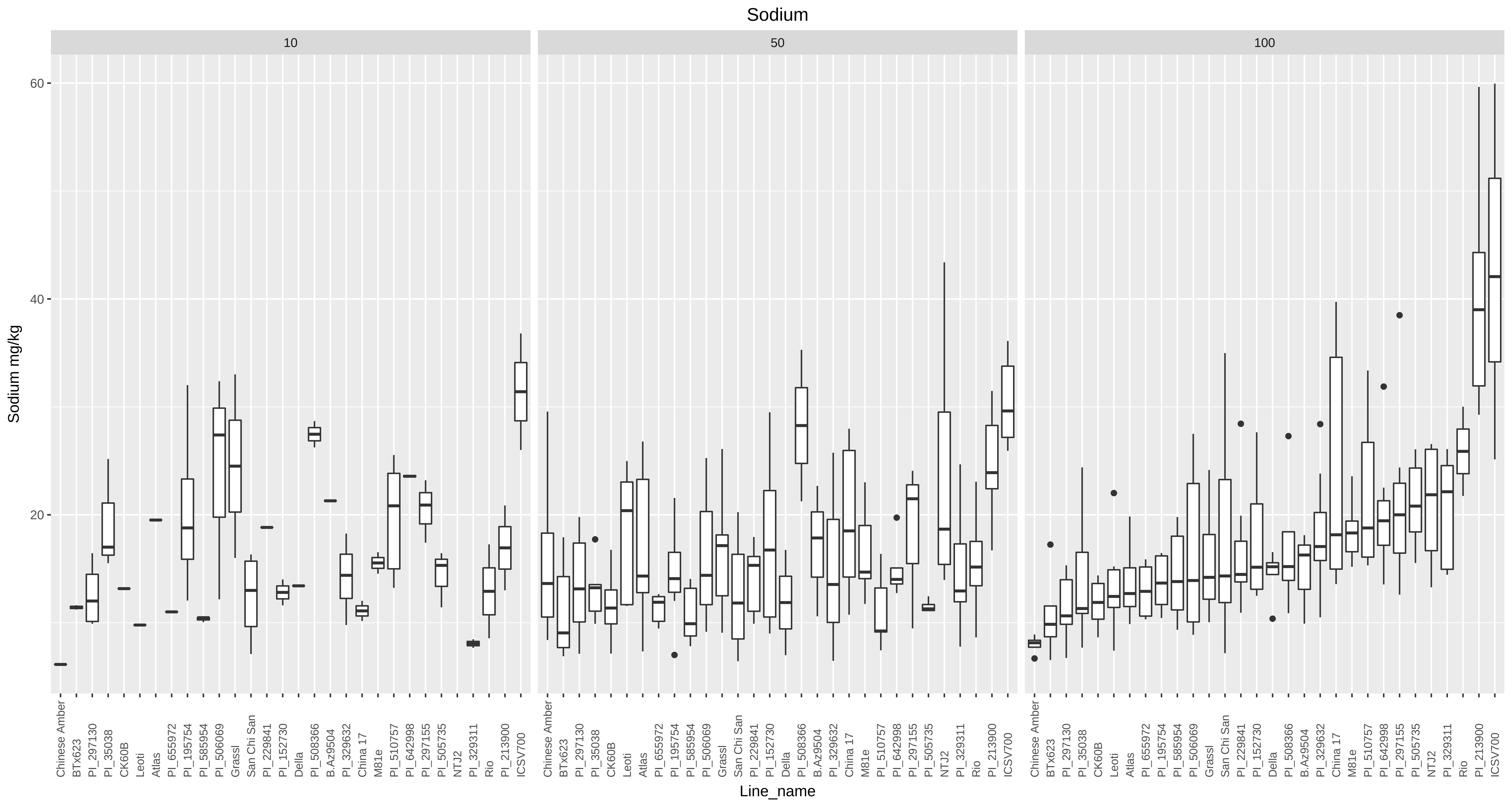

Sodium
10
50
100
Sodium mg/kg
60
40
20
Line_name
Chinese Amber
BTx623
PI_297130
PI_35038
CK60B
Leoti
Atlas
PI_655972
PI_195754
PI_585954
PI_506069
Grassl
San Chi San
PI_229841
PI_152730
Della
PI_508366
B.Az9504
PI_329632
China 17
M81e
PI_510757
PI_642998
PI_297155
PI_505735
NTJ2
PI_329311
Rio
PI_213900
ICSV700

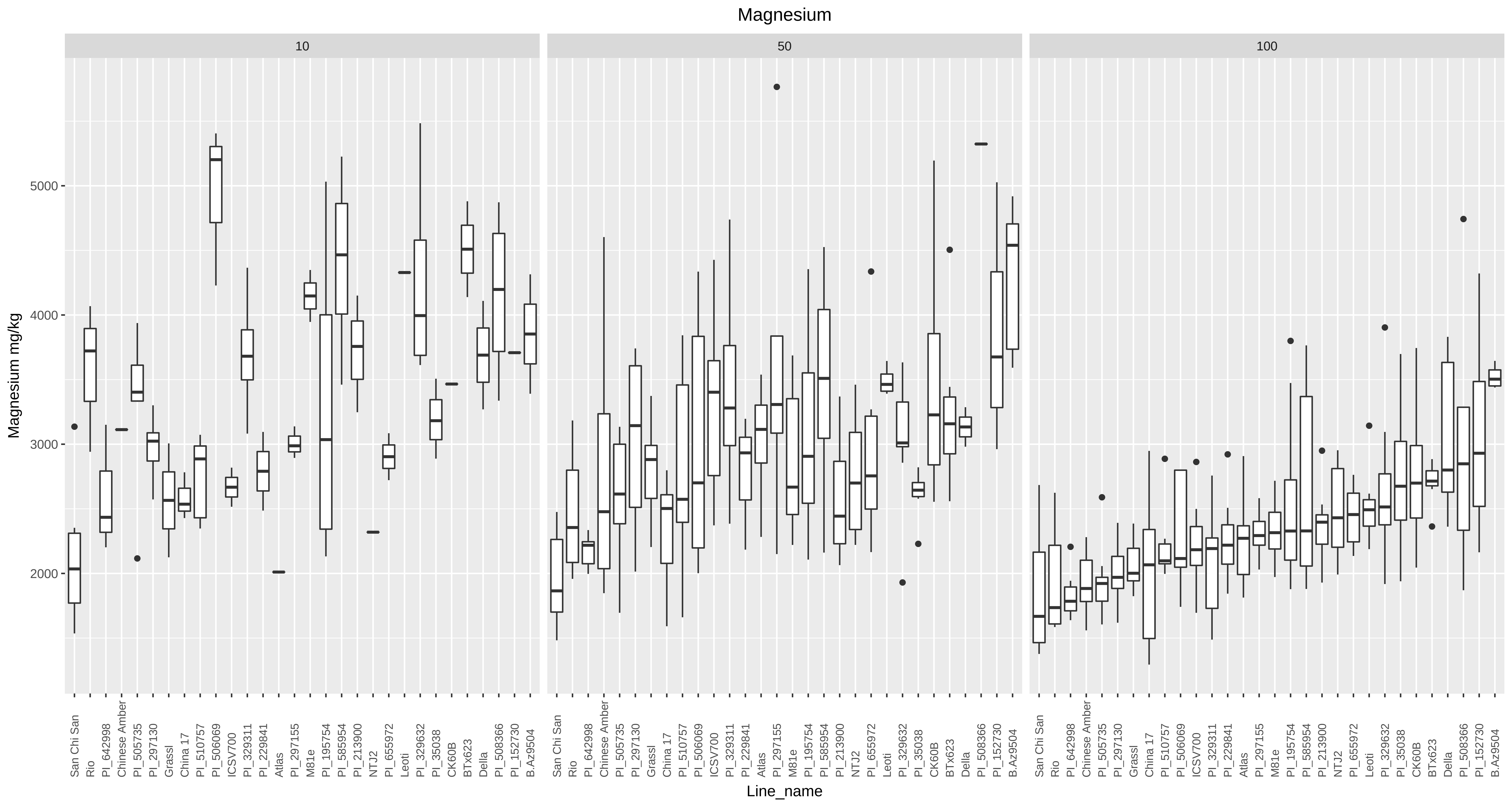

Magnesium
10
50
100
Magnesium mg/kg
5000
4000
3000
2000
Line_name
San Chi San
Rio
PI_642998
Chinese Amber
PI_505735
PI_297130
Grassl
China 17
PI_510757
PI_506069
ICSV700
PI_329311
PI_229841
Atlas
PI_297155
M81e
PI_195754
PI_585954
PI_213900
NTJ2
PI_655972
Leoti
PI_329632
PI_35038
CK60B
BTx623
Della
PI_508366
PI_152730
B.Az9504

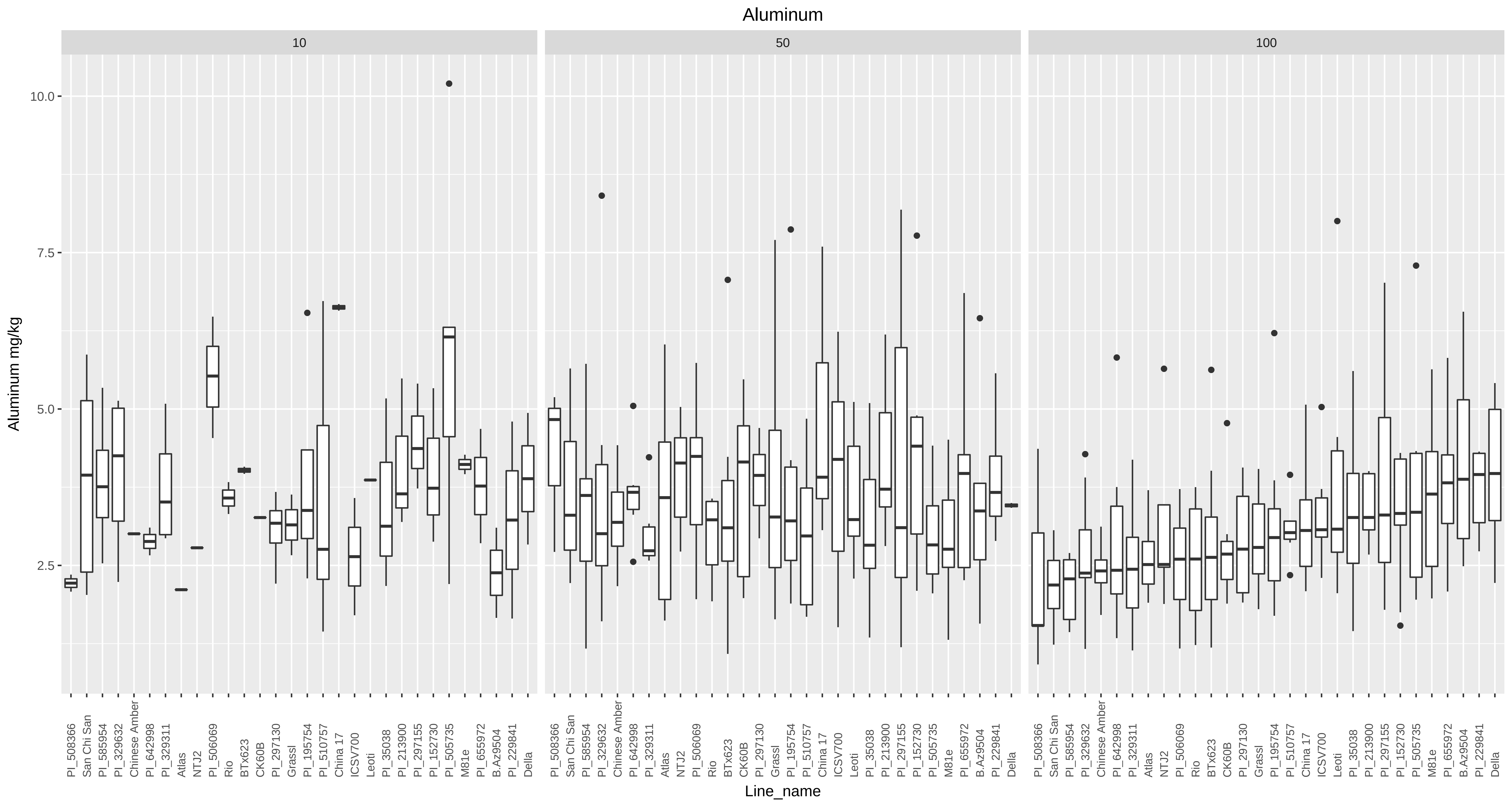

Aluminum
10
50
100
Aluminum mg/kg
10.0
7.5
5.0
2.5
Line_name
PI_508366
San Chi San
PI_585954
PI_329632
Chinese Amber
PI_642998
PI_329311
Atlas
NTJ2
PI_506069
Rio
BTx623
CK60B
PI_297130
Grassl
PI_195754
PI_510757
China 17
ICSV700
Leoti
PI_35038
PI_213900
PI_297155
PI_152730
PI_505735
M81e
PI_655972
B.Az9504
PI_229841
Della

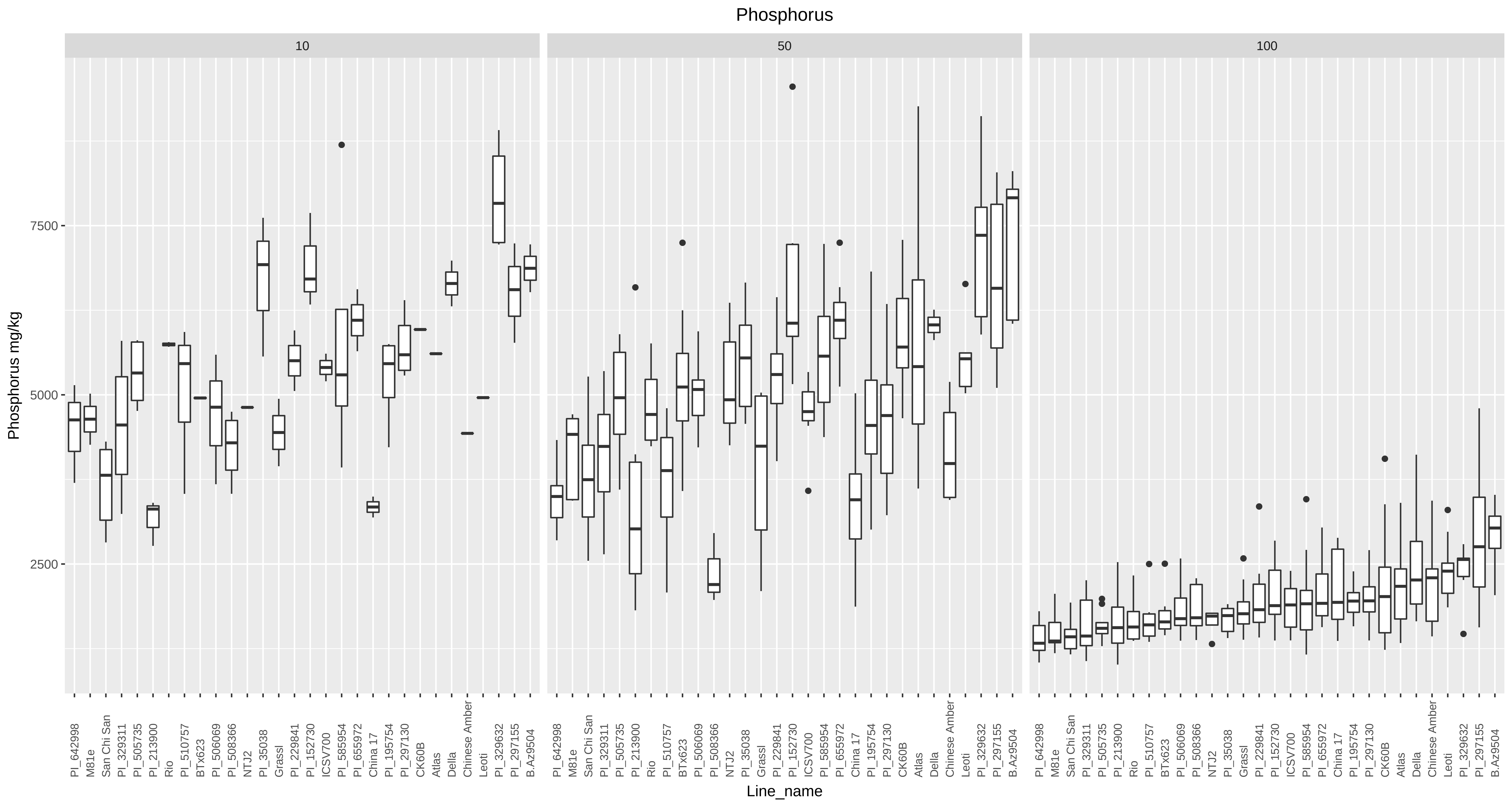

Phosphorus
10
50
100
Phosphorus mg/kg
7500
5000
2500
Line_name
PI_642998
M81e
San Chi San
PI_329311
PI_505735
PI_213900
Rio
PI_510757
BTx623
PI_506069
PI_508366
NTJ2
PI_35038
Grassl
PI_229841
PI_152730
ICSV700
PI_585954
PI_655972
China 17
PI_195754
PI_297130
CK60B
Atlas
Della
Chinese Amber
Leoti
PI_329632
PI_297155
B.Az9504

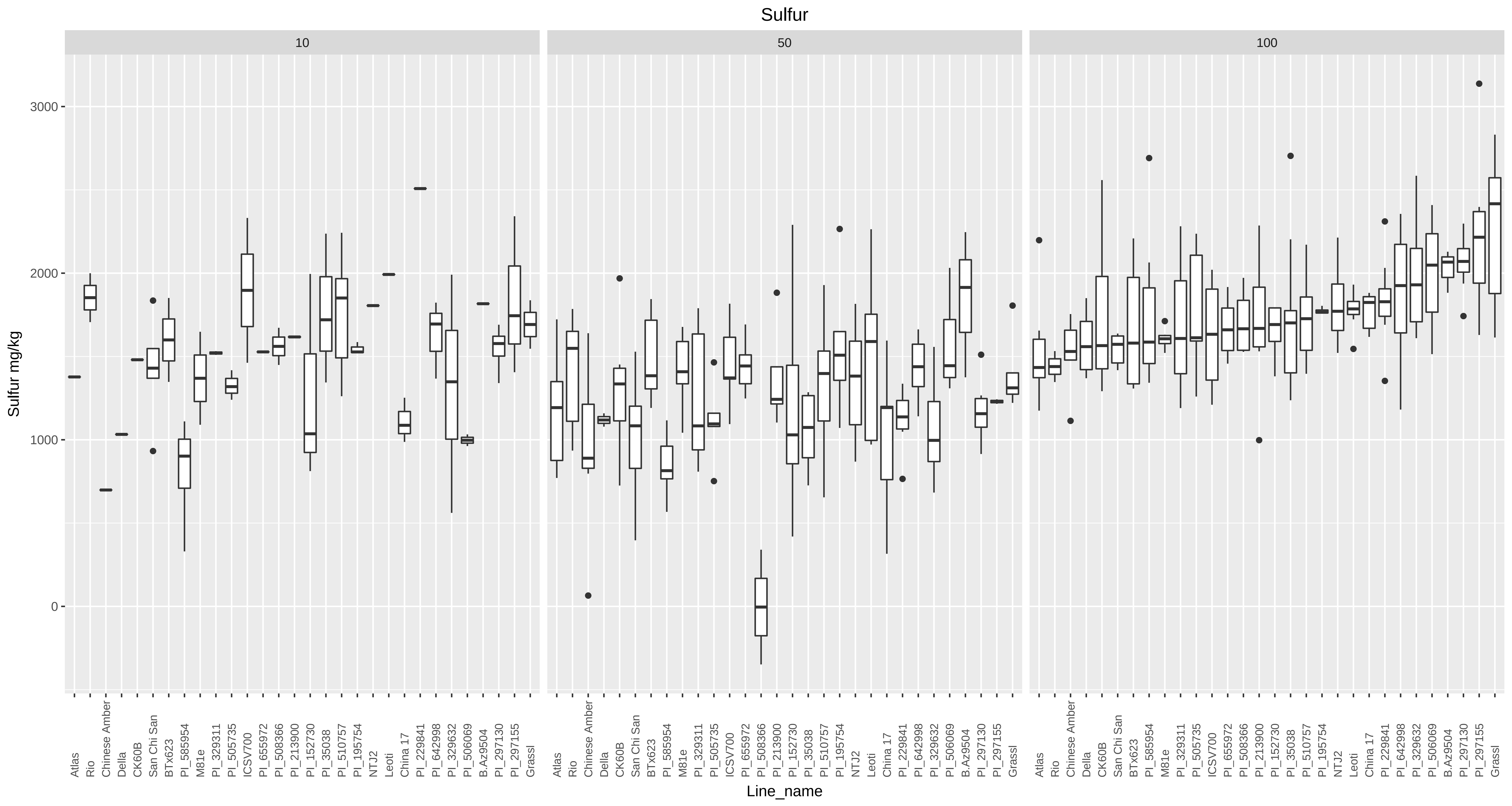

Sulfur
10
50
100
Sulfur mg/kg
3000
2000
1000
0
Line_name
Atlas
Rio
Chinese Amber
Della
CK60B
San Chi San
BTx623
PI_585954
M81e
PI_329311
PI_505735
ICSV700
PI_655972
PI_508366
PI_213900
PI_152730
PI_35038
PI_510757
PI_195754
NTJ2
Leoti
China 17
PI_229841
PI_642998
PI_329632
PI_506069
B.Az9504
PI_297130
PI_297155
Grassl

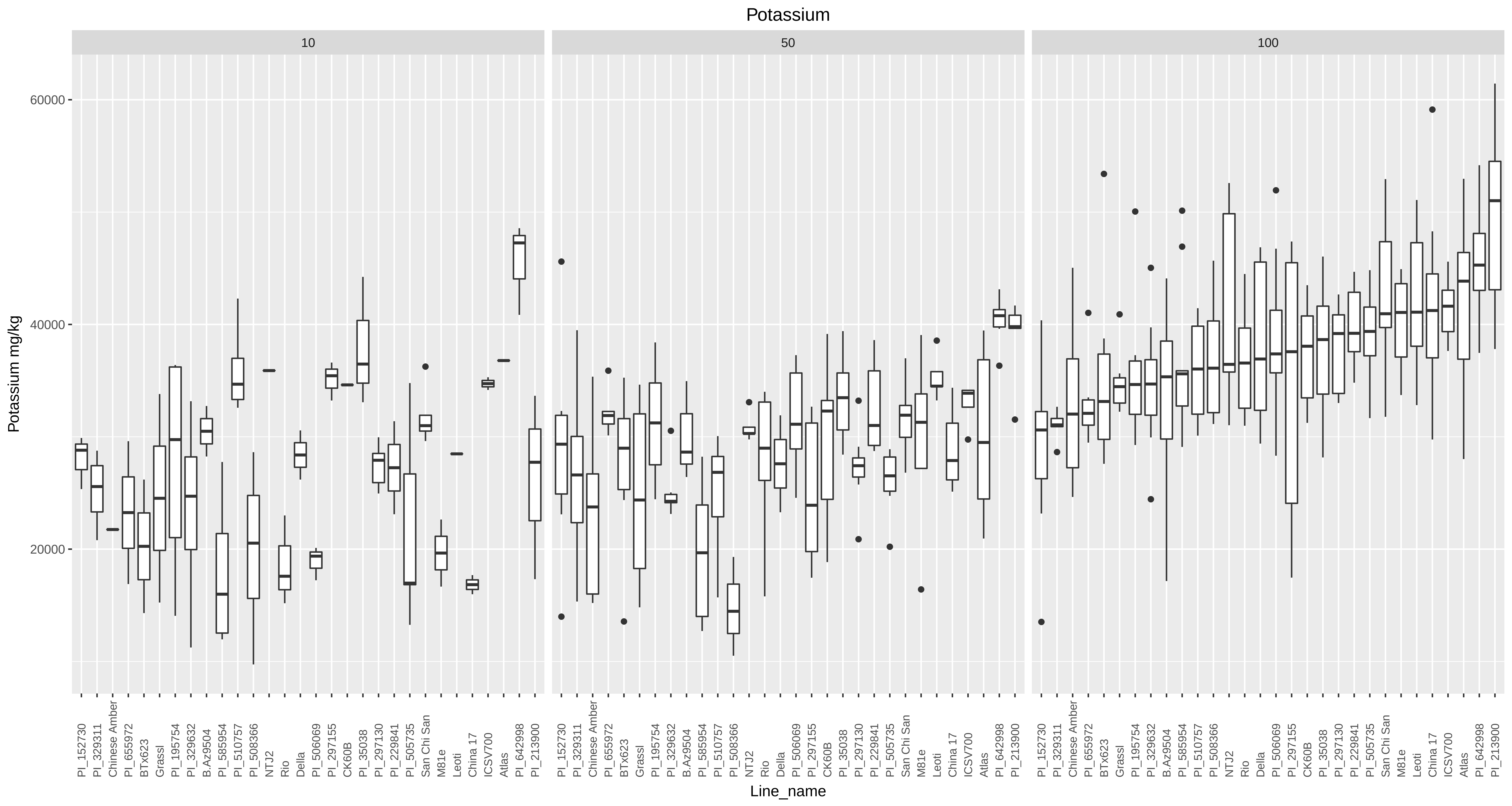
Potassium
10
50
100
Potassium mg/kg
60000
40000
20000
Line_name
PI_152730
PI_329311
Chinese Amber
PI_655972
BTx623
Grassl
PI_195754
PI_329632
B.Az9504
PI_585954
PI_510757
PI_508366
NTJ2
Rio
Della
PI_506069
PI_297155
CK60B
PI_35038
PI_297130
PI_229841
PI_505735
San Chi San
M81e
Leoti
China 17
ICSV700
Atlas
PI_642998
PI_213900

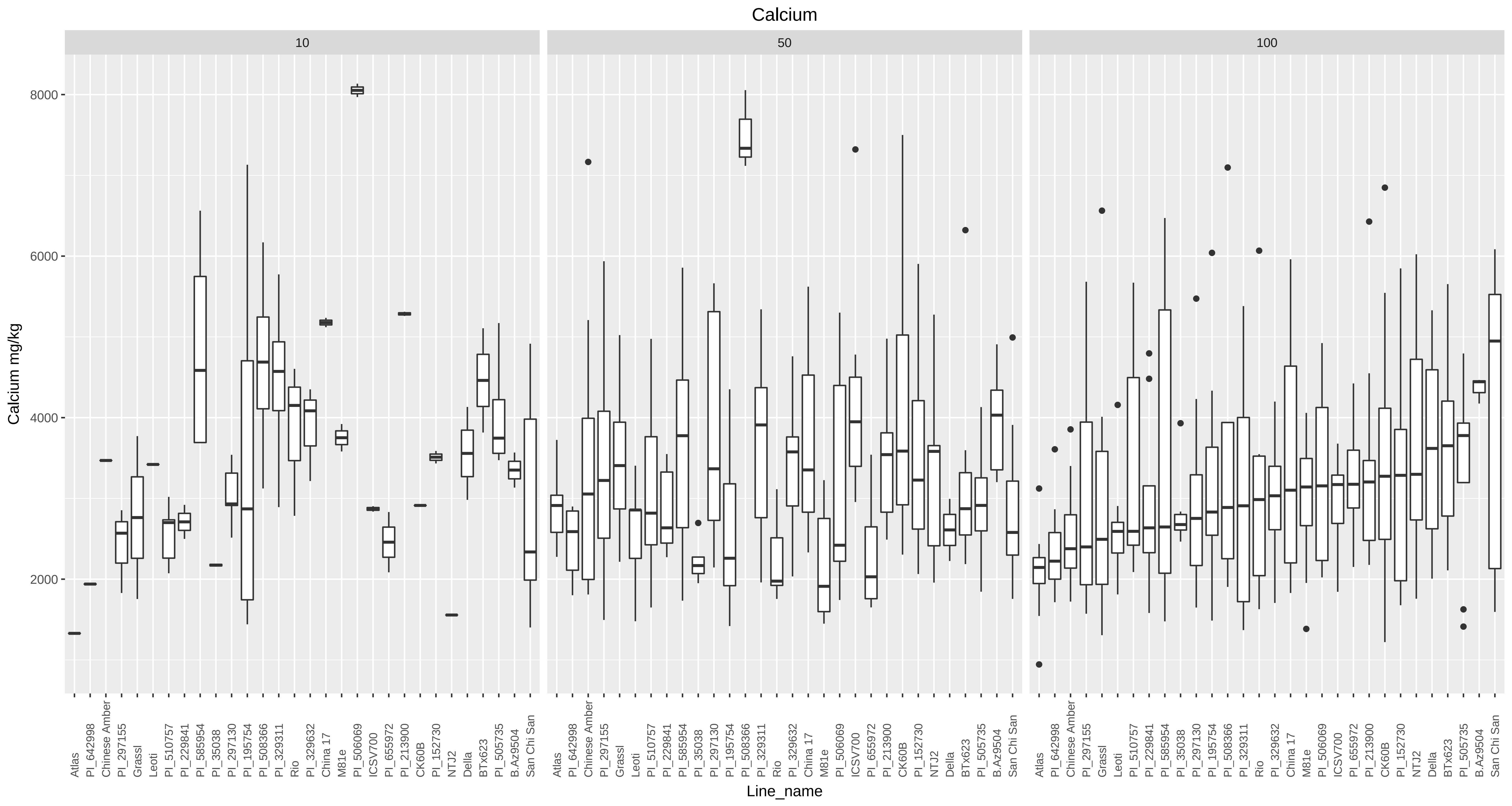
Calcium
10
50
100
Calcium mg/kg
8000
6000
4000
2000
Line_name
Atlas
PI_642998
Chinese Amber
PI_297155
Grassl
Leoti
PI_510757
PI_229841
PI_585954
PI_35038
PI_297130
PI_195754
PI_508366
PI_329311
Rio
PI_329632
China 17
M81e
PI_506069
ICSV700
PI_655972
PI_213900
CK60B
PI_152730
NTJ2
Della
BTx623
PI_505735
B.Az9504
San Chi San

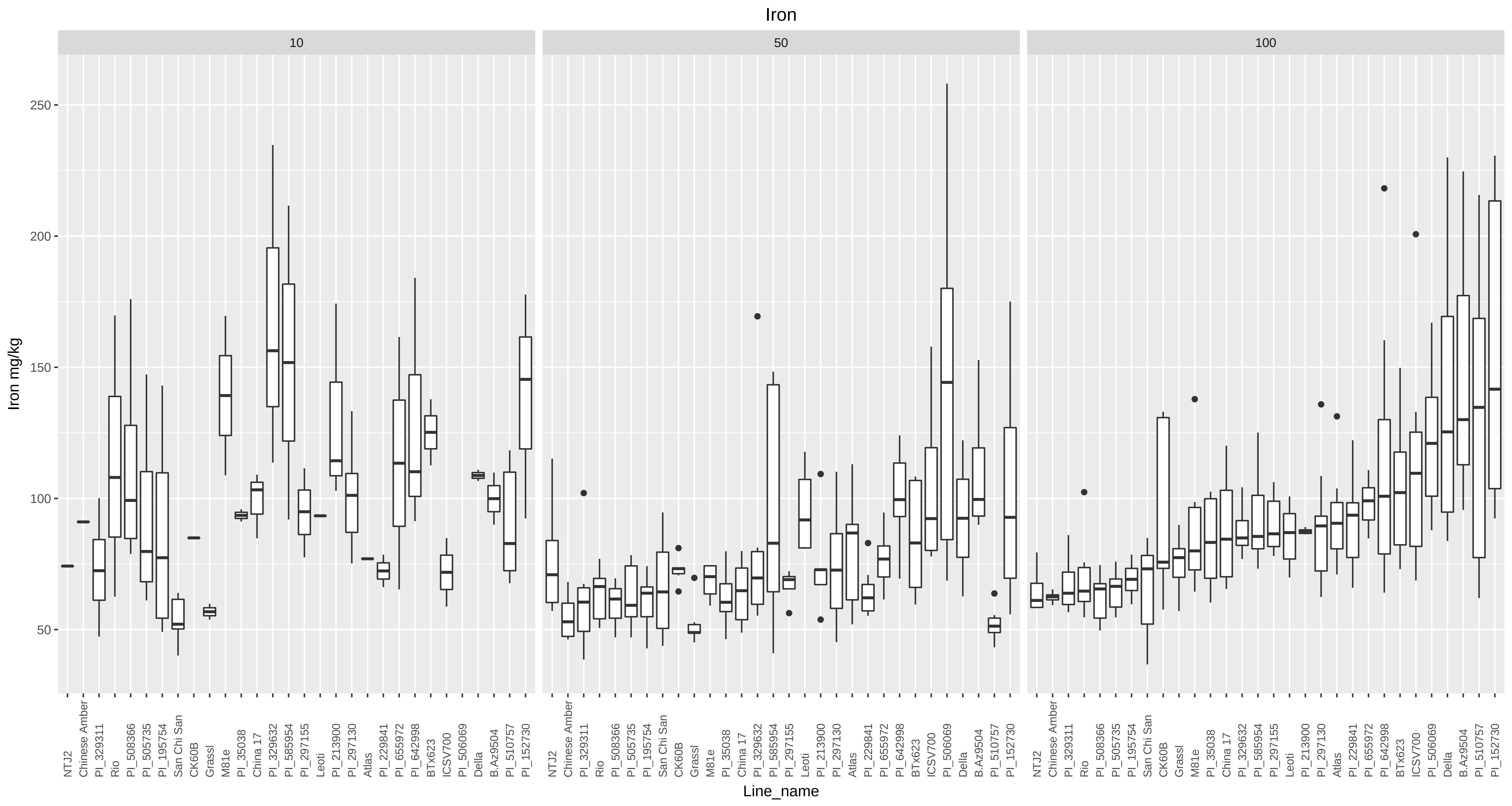
Iron
10
50
100
Iron mg/kg
250
200
150
100
50
NTJ2
Chinese Amber
PI_329311
Rio
PI_508366
PI_505735
PI_195754
San Chi San
CK60B
Grassl
M81e
PI_35038
China 17
PI_329632
PI_585954
PI_297155
Leoti
PI_213900
PI_297130
Atlas
PI_229841
PI_655972
PI_642998
BTx623
ICSV700
PI_506069
Della
B.Az9504
PI_510757
PI_152730
Line_name

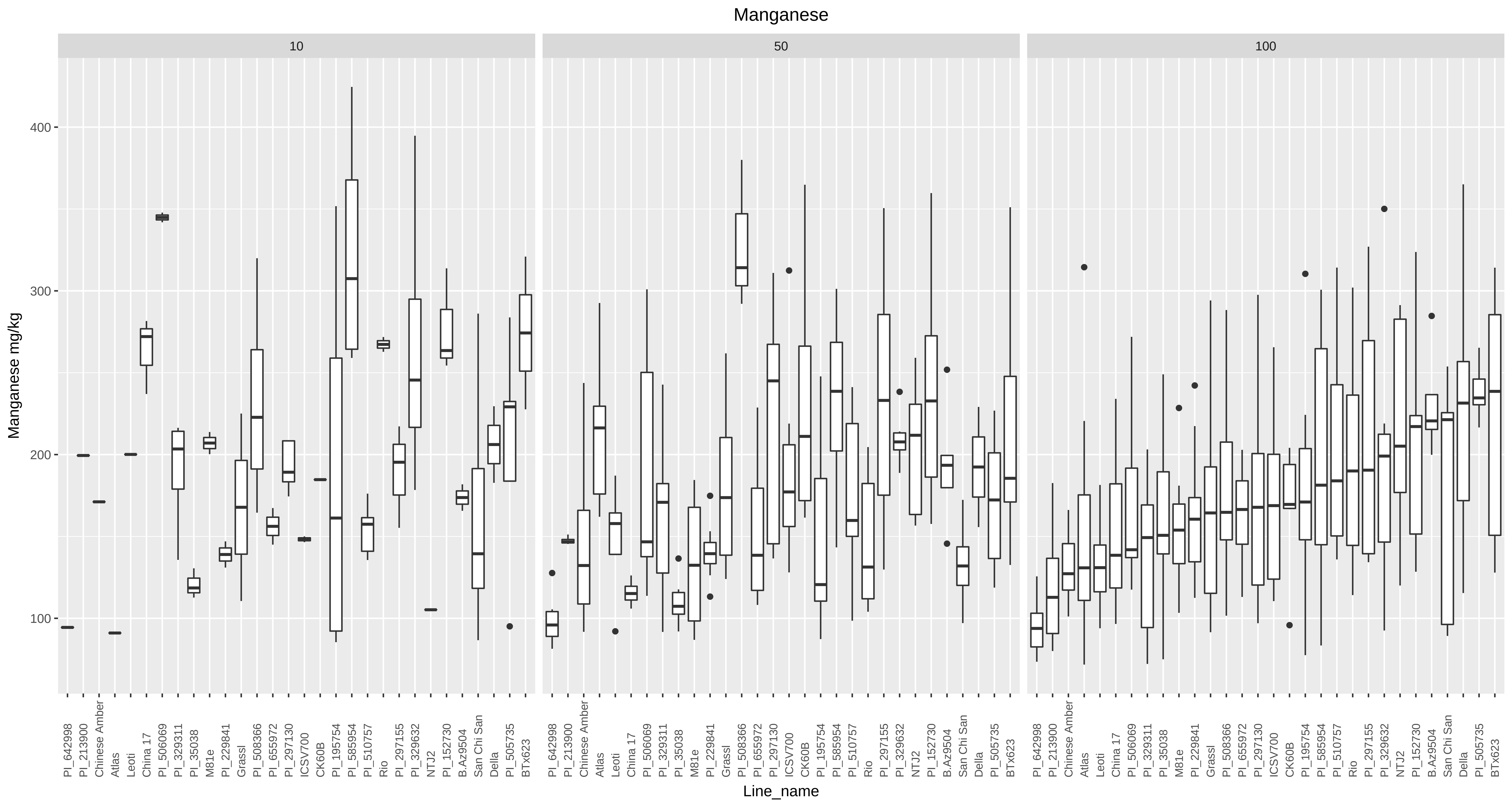
Manganese
10
50
100
Manganese mg/kg
400
300
200
100
Line_name
PI_642998
PI_213900
Chinese Amber
Atlas
Leoti
China 17
PI_506069
PI_329311
PI_35038
M81e
PI_229841
Grassl
PI_508366
PI_655972
PI_297130
ICSV700
CK60B
PI_195754
PI_585954
PI_510757
Rio
PI_297155
PI_329632
NTJ2
PI_152730
B.Az9504
San Chi San
Della
PI_505735
BTx623

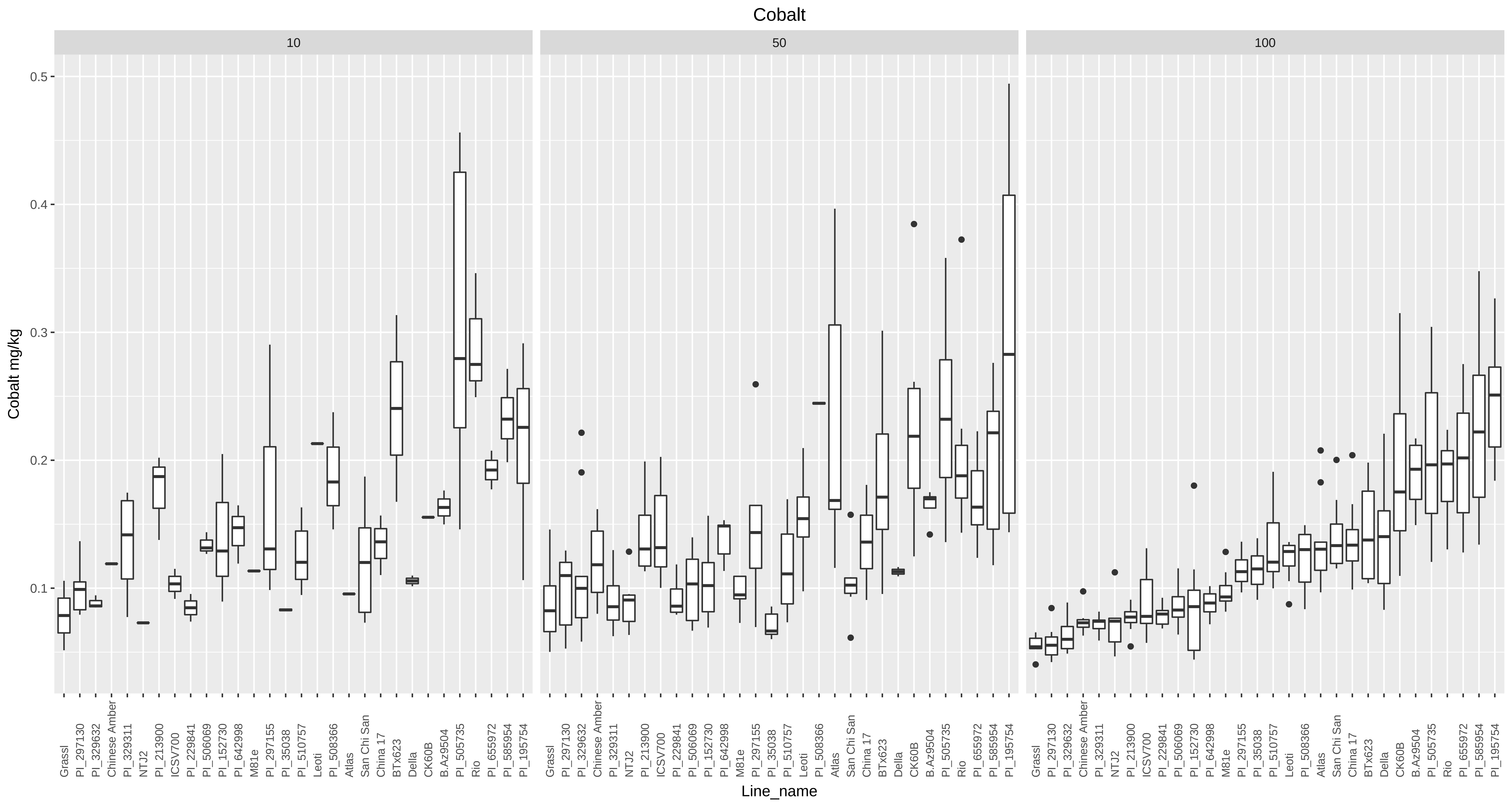
Cobalt
10
50
100
Cobalt mg/kg
0.1
0.2
0.3
0.4
0.5
Line_name
Grassl
PI_297130
PI_329632
Chinese Amber
PI_329311
NTJ2
PI_213900
ICSV700
PI_229841
PI_506069
PI_152730
PI_642998
M81e
PI_297155
PI_35038
PI_510757
Leoti
PI_508366
Atlas
San Chi San
China 17
BTx623
Della
CK60B
B.Az9504
PI_505735
Rio
PI_655972
PI_585954
PI_195754

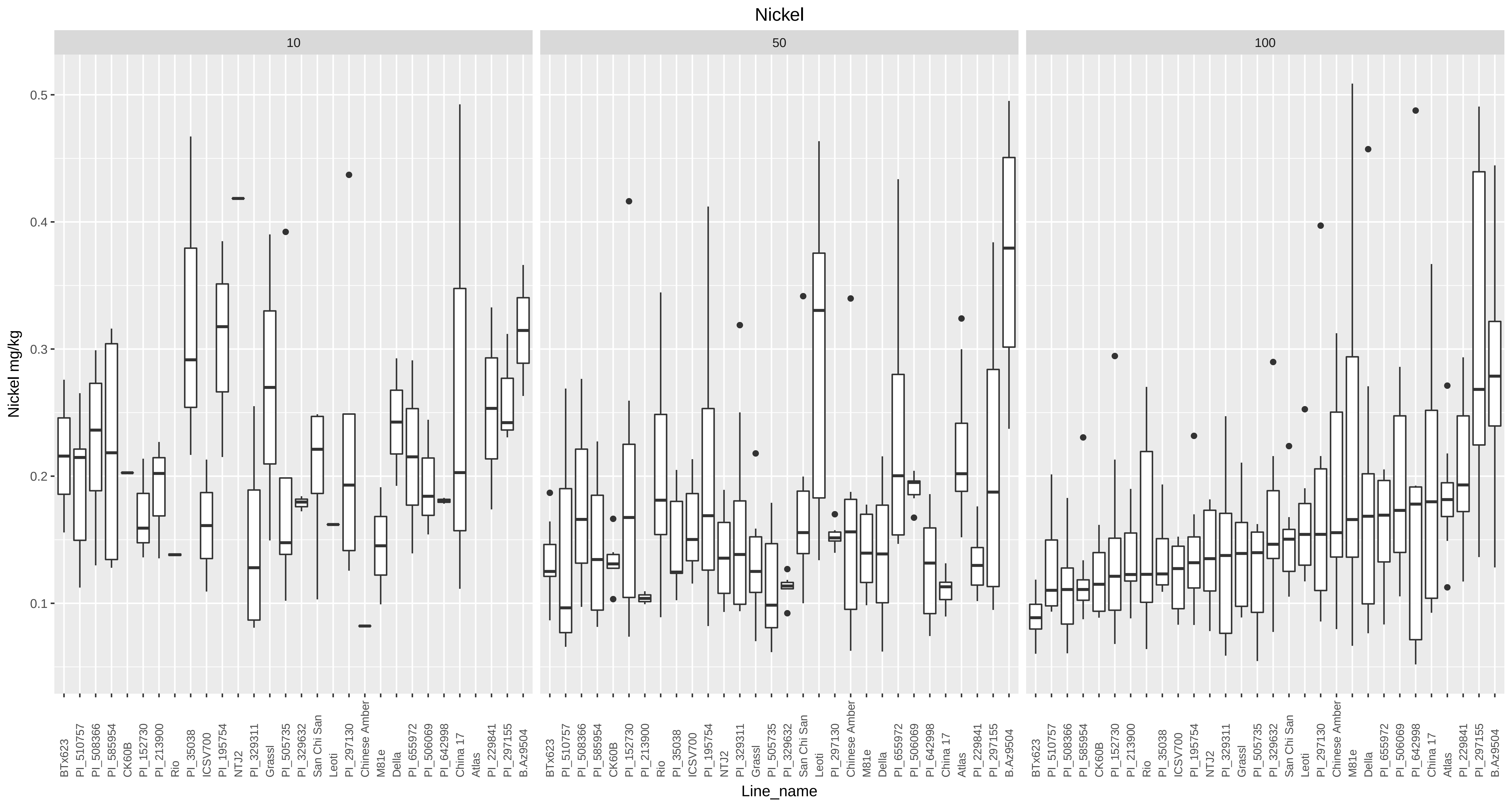

Nickel
10
50
100
Nickel mg/kg
0.5
0.4
0.3
0.2
0.1
Line_name
BTx623
PI_510757
PI_508366
PI_585954
CK60B
PI_152730
PI_213900
Rio
PI_35038
ICSV700
PI_195754
NTJ2
PI_329311
Grassl
PI_505735
PI_329632
San Chi San
Leoti
PI_297130
Chinese Amber
M81e
Della
PI_655972
PI_506069
PI_642998
China 17
Atlas
PI_229841
PI_297155
B.Az9504

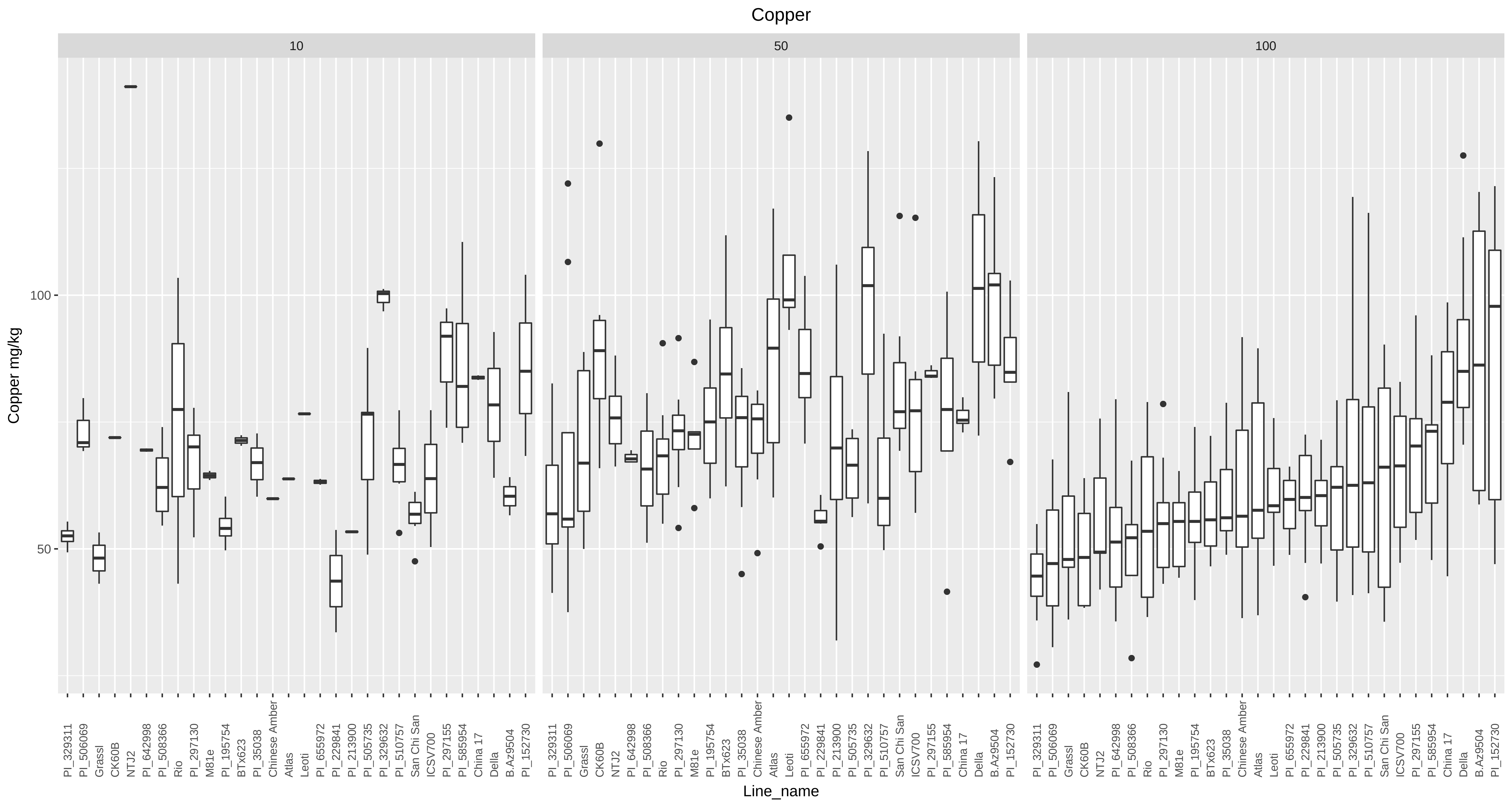

Copper
10
50
100
Copper mg/kg
100
50
Line_name
PI_329311
PI_506069
Grassl
CK60B
NTJ2
PI_642998
PI_508366
Rio
PI_297130
M81e
PI_195754
BTx623
PI_35038
Chinese Amber
Atlas
Leoti
PI_655972
PI_229841
PI_213900
PI_505735
PI_329632
PI_510757
San Chi San
ICSV700
PI_297155
PI_585954
China 17
Della
B.Az9504
PI_152730

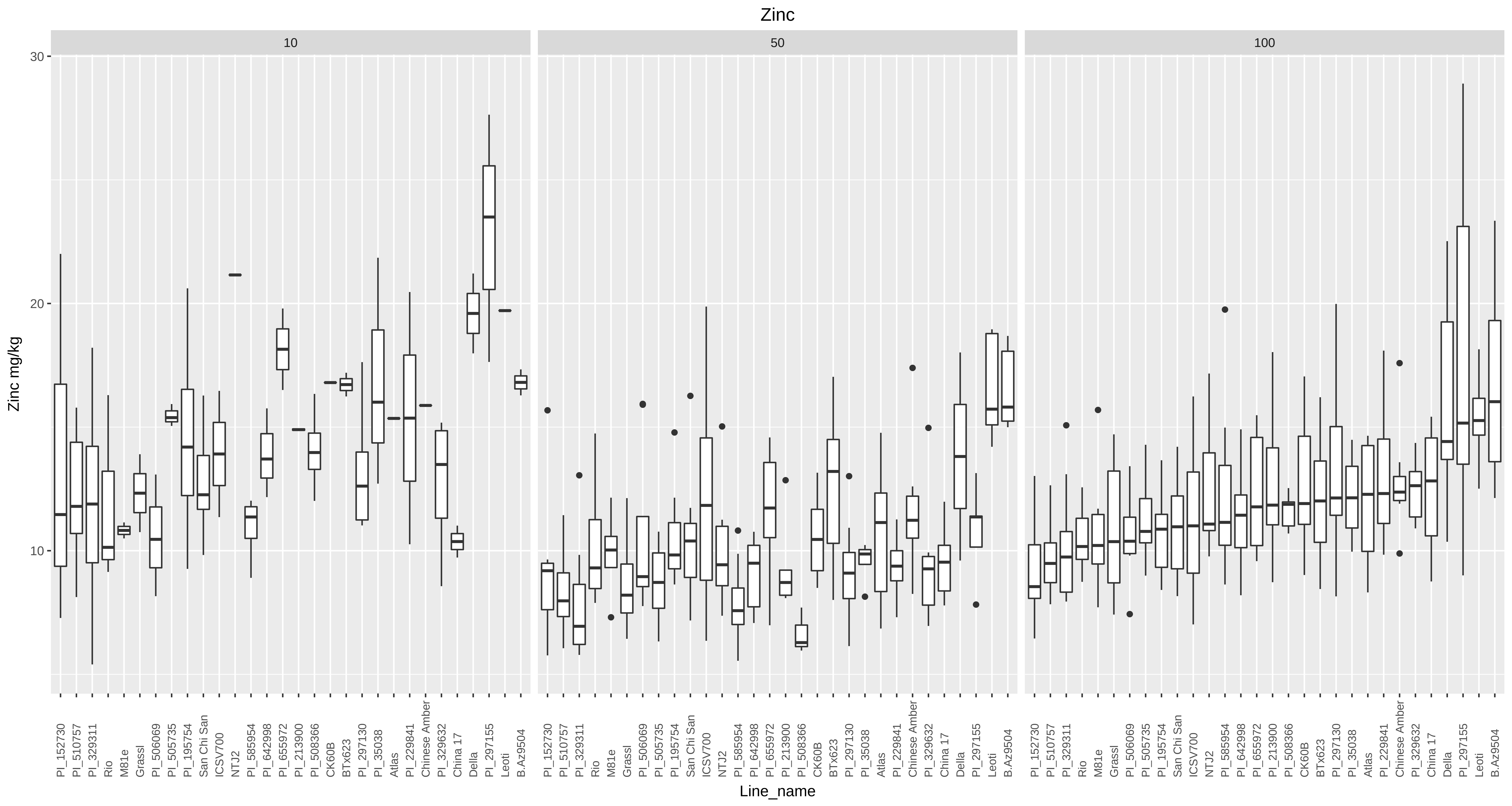

Zinc
10
50
100
Zinc mg/kg
30
20
10
Line_name
PI_152730
PI_510757
PI_329311
Rio
M81e
Grassl
PI_506069
PI_505735
PI_195754
San Chi San
ICSV700
NTJ2
PI_585954
PI_642998
PI_655972
PI_213900
PI_508366
CK60B
BTx623
PI_297130
PI_35038
Atlas
PI_229841
Chinese Amber
PI_329632
China 17
Della
PI_297155
Leoti
B.Az9504

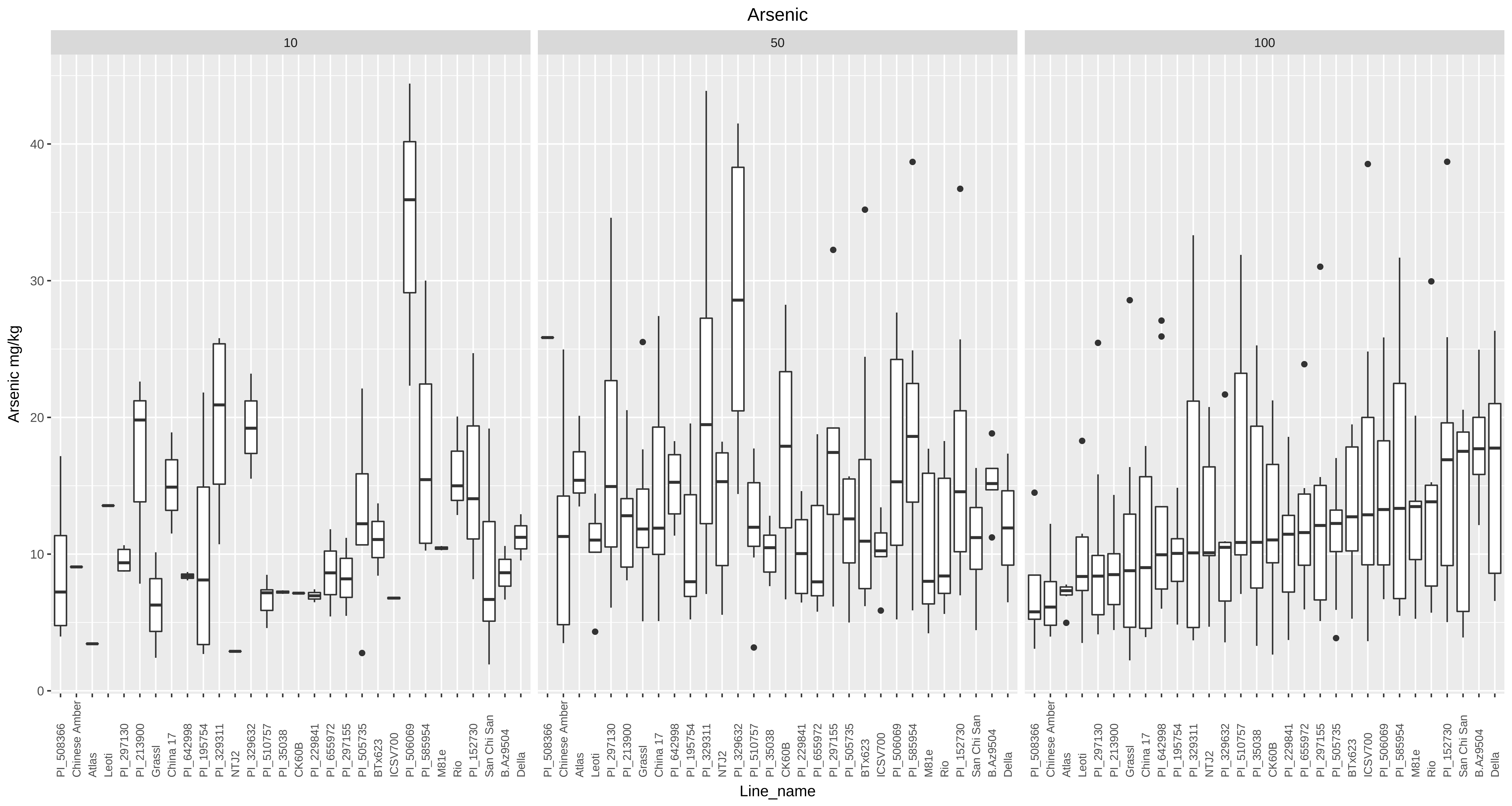

Arsenic
10
50
100
Arsenic mg/kg
0
10
20
30
40
Line_name
PI_508366
Chinese Amber
Atlas
Leoti
PI_297130
PI_213900
Grassl
China 17
PI_642998
PI_195754
PI_329311
NTJ2
PI_329632
PI_510757
PI_35038
CK60B
PI_229841
PI_655972
PI_297155
PI_505735
BTx623
ICSV700
PI_506069
PI_585954
M81e
Rio
PI_152730
San Chi San
B.Az9504
Della

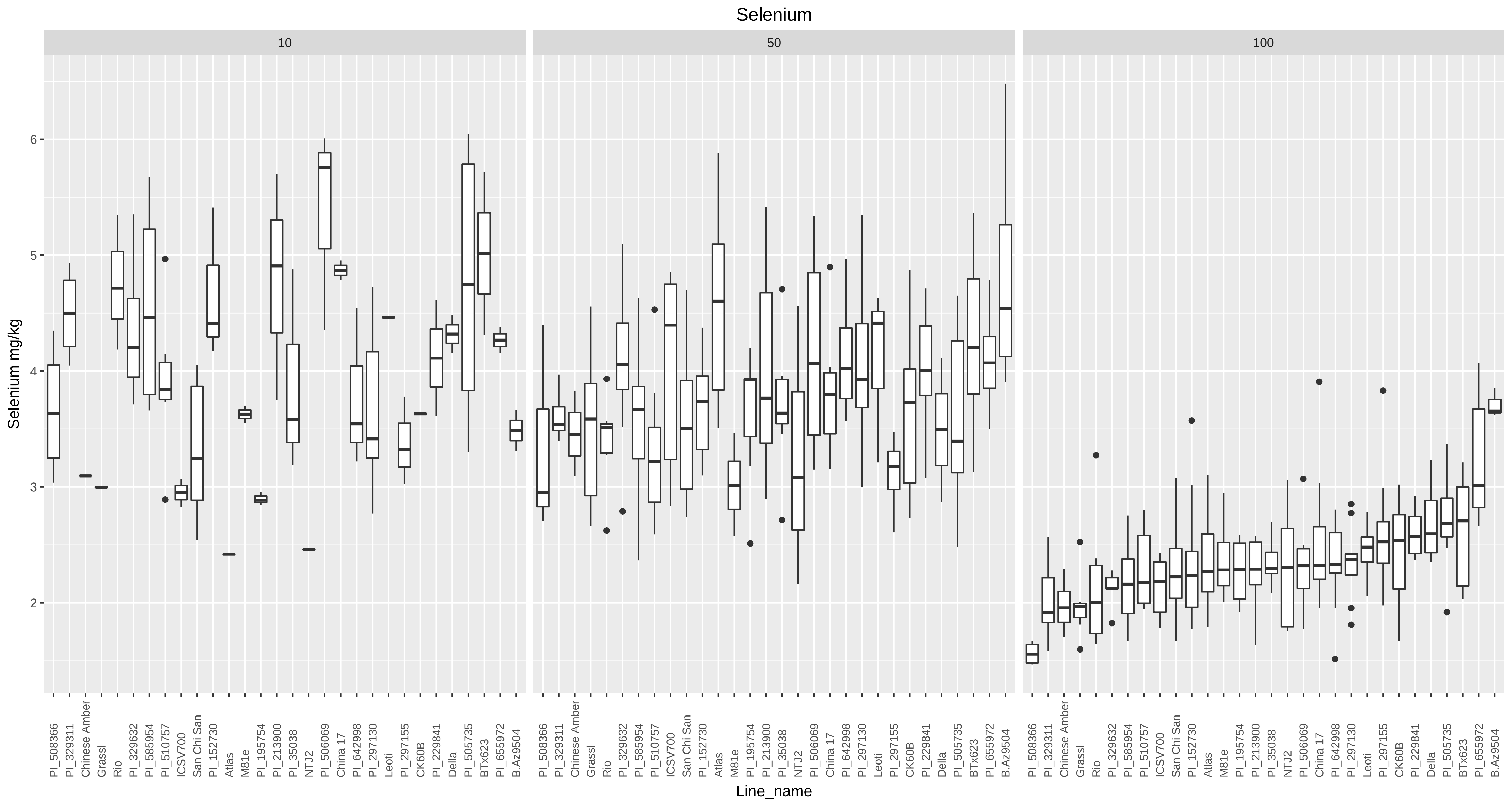

Selenium
10
50
100
Selenium mg/kg
Line_name
PI_508366
PI_329311
Chinese Amber
Grassl
Rio
PI_329632
PI_585954
PI_510757
ICSV700
San Chi San
PI_152730
Atlas
M81e
PI_195754
PI_213900
PI_35038
NTJ2
PI_506069
China 17
PI_642998
PI_297130
Leoti
PI_297155
CK60B
PI_229841
Della
PI_505735
BTx623
PI_655972
B.Az9504

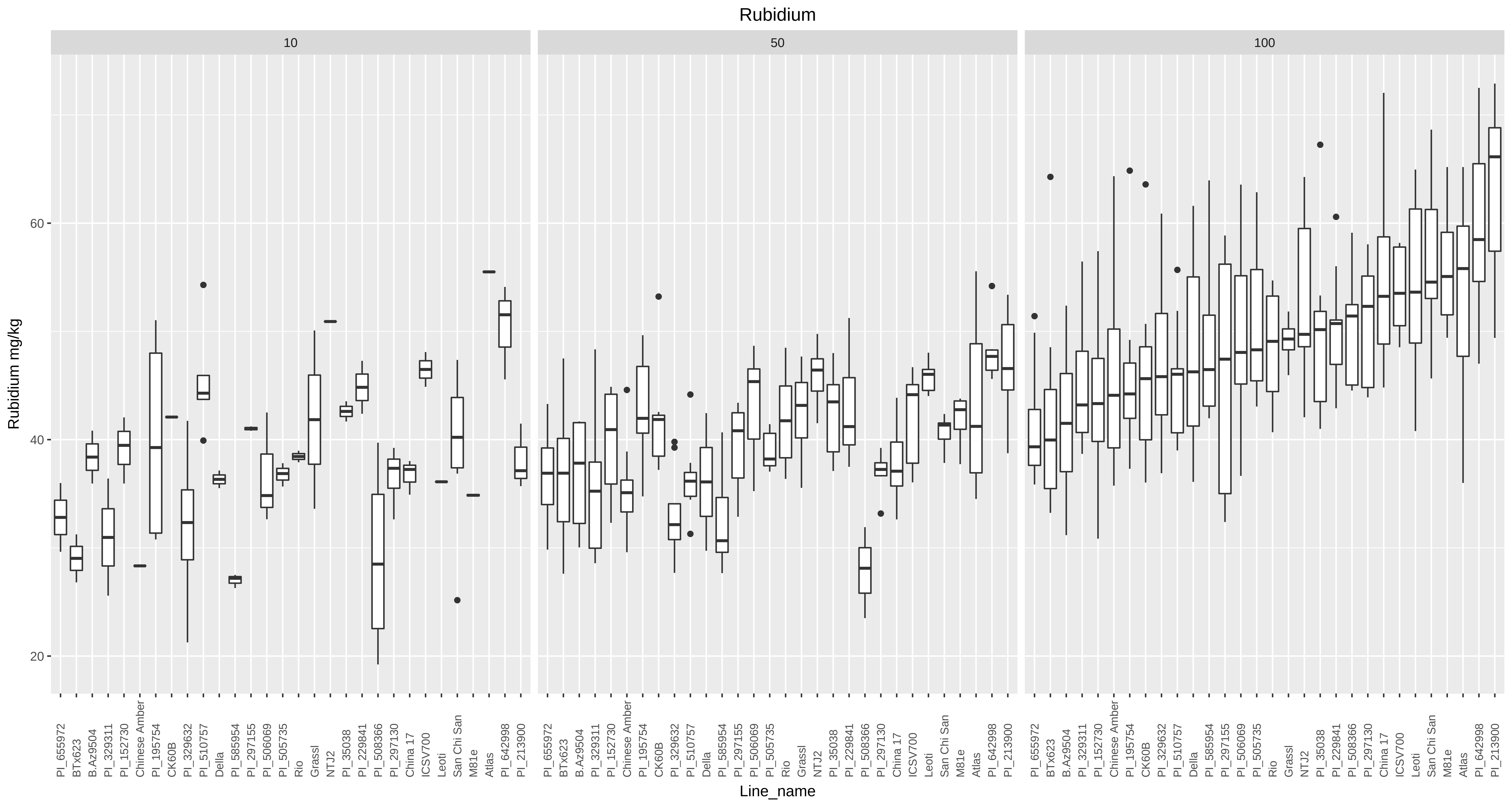
Rubidium
10
50
100
Rubidium mg/kg
60
40
20
Line_name
PI_655972
BTx623
B.Az9504
PI_329311
PI_152730
Chinese Amber
PI_195754
CK60B
PI_329632
PI_510757
Della
PI_585954
PI_297155
PI_506069
PI_505735
Rio
Grassl
NTJ2
PI_35038
PI_229841
PI_508366
PI_297130
China 17
ICSV700
Leoti
San Chi San
M81e
Atlas
PI_642998
PI_213900

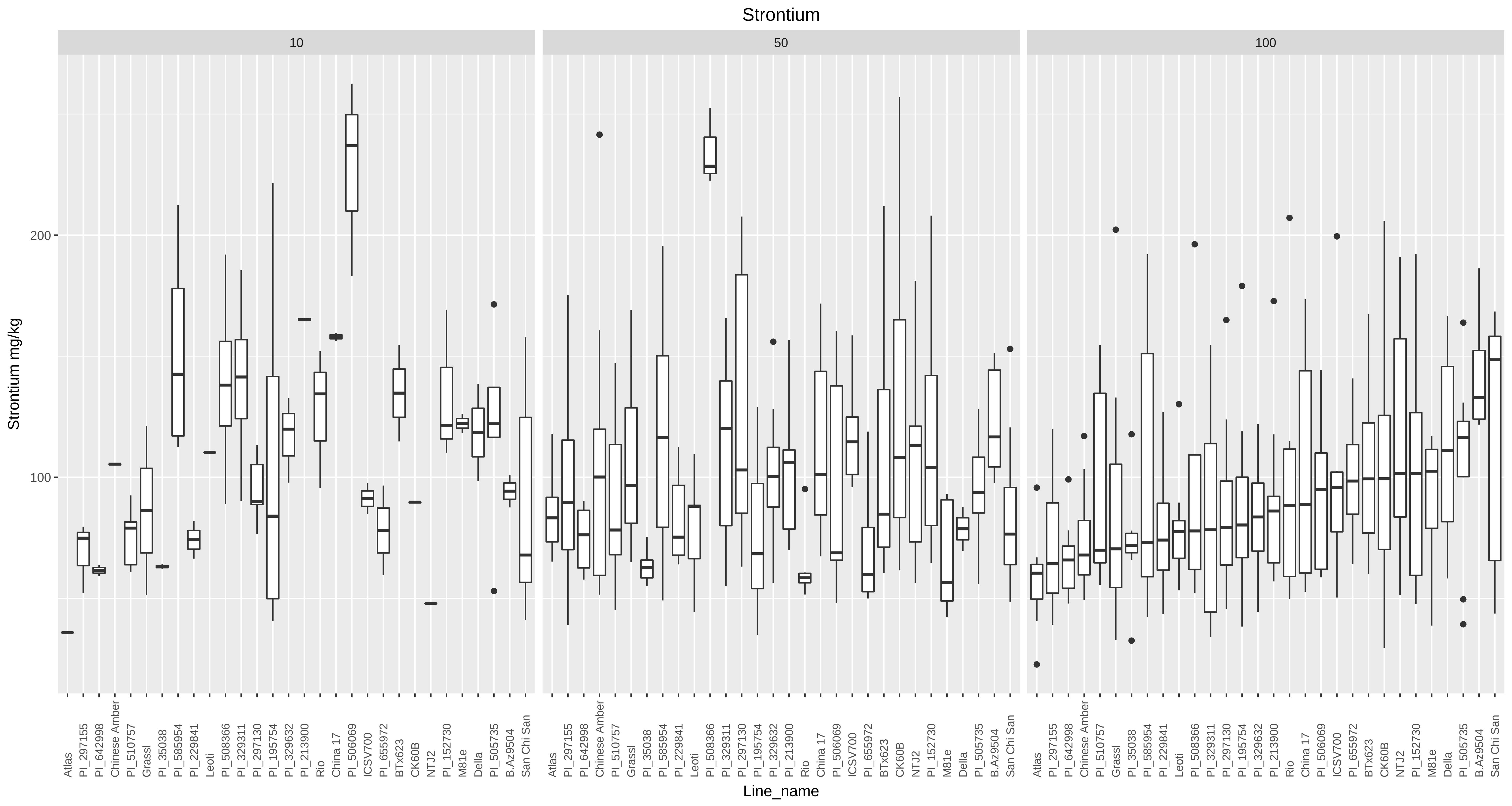

Strontium
10
50
100
Strontium mg/kg
200
100
Line_name
Atlas
PI_297155
PI_642998
Chinese Amber
PI_510757
Grassl
PI_35038
PI_585954
PI_229841
Leoti
PI_508366
PI_329311
PI_297130
PI_195754
PI_329632
PI_213900
Rio
China 17
PI_506069
ICSV700
PI_655972
BTx623
CK60B
NTJ2
PI_152730
M81e
Della
PI_505735
B.Az9504
San Chi San

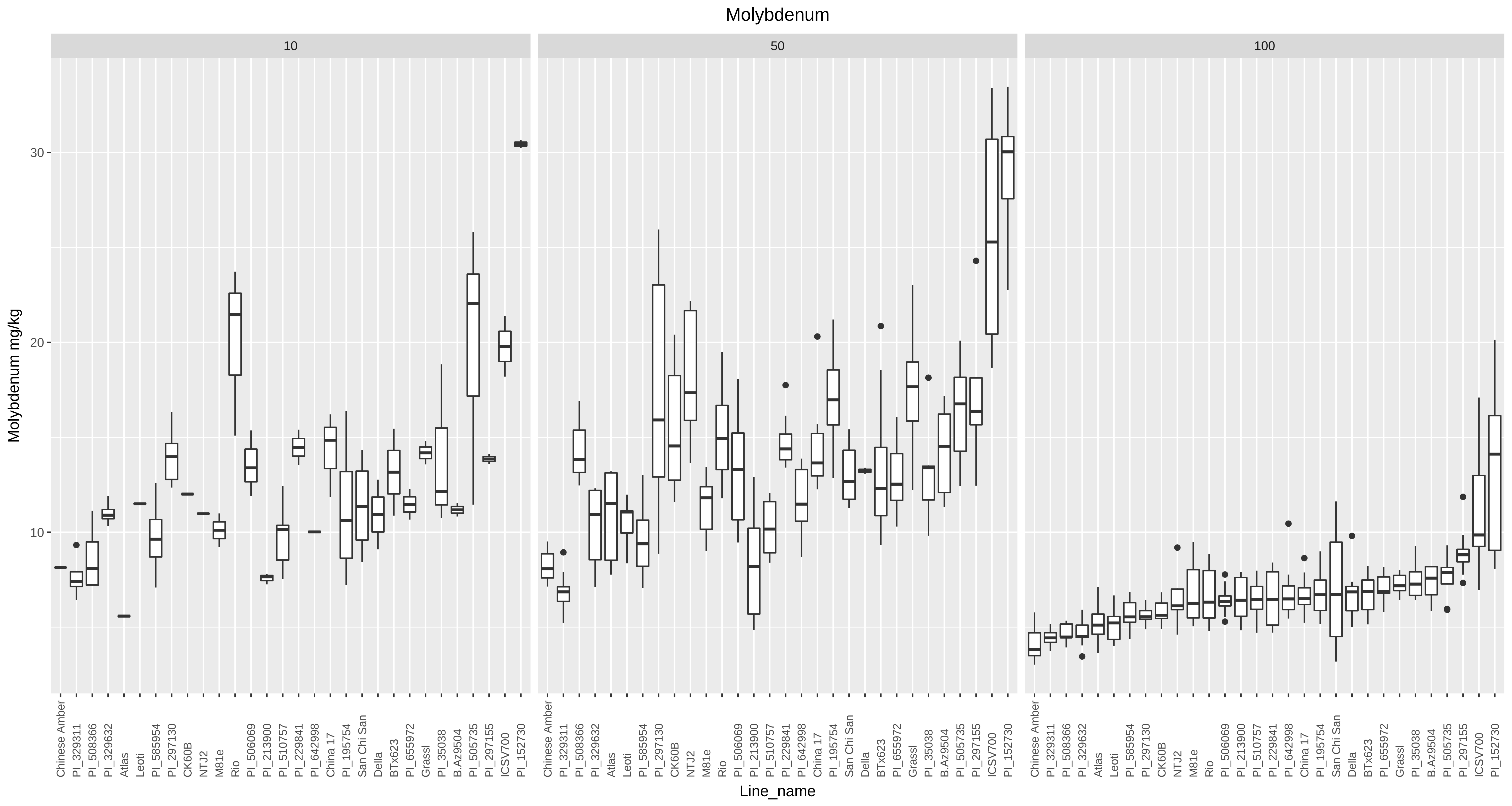

Molybdenum
10
50
100
Molybdenum mg/kg
30
20
10
Line_name
Chinese Amber
PI_329311
PI_508366
PI_329632
Atlas
Leoti
PI_585954
PI_297130
CK60B
NTJ2
M81e
Rio
PI_506069
PI_213900
PI_510757
PI_229841
PI_642998
China 17
PI_195754
San Chi San
Della
BTx623
PI_655972
Grassl
PI_35038
B.Az9504
PI_505735
PI_297155
ICSV700
PI_152730

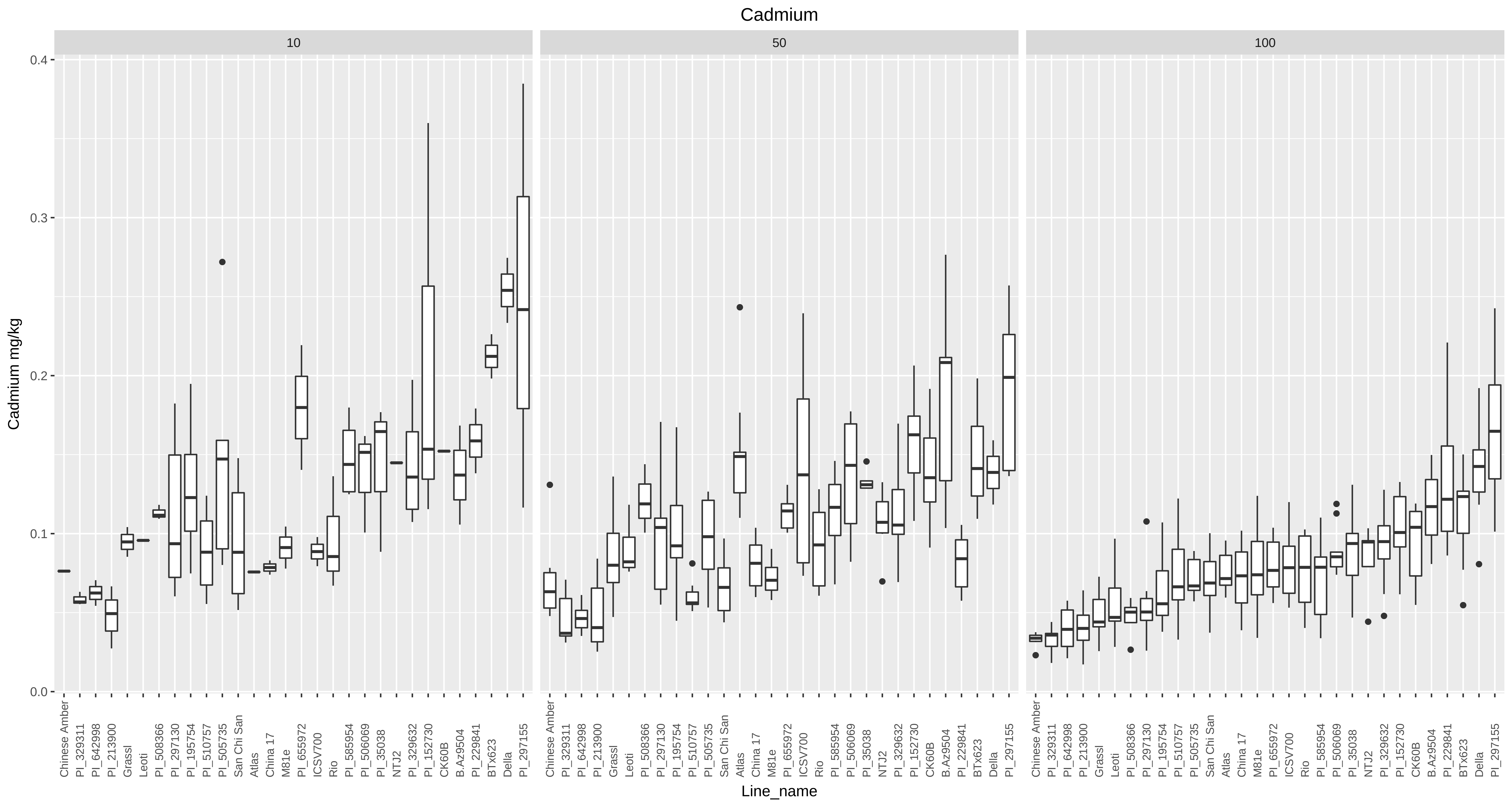

Cadmium
10
50
100
Cadmium mg/kg
0.0
0.1
0.2
0.3
0.4
Chinese Amber
PI_329311
PI_642998
PI_213900
Grassl
Leoti
PI_508366
PI_297130
PI_195754
PI_510757
PI_505735
San Chi San
Atlas
China 17
M81e
PI_655972
ICSV700
Rio
PI_585954
PI_506069
PI_35038
NTJ2
PI_329632
PI_152730
CK60B
B.Az9504
PI_229841
BTx623
Della
PI_297155
Line_name
