# Supplementary material for: High‐throughput profiling and analysis of plant responses over time to abiotic stress
Source: Plant Direct. 2017 Oct 25;1(4):e00023. doi: 10.1002/pld3.23 (PMC6508565; doi:10.1002/pld3.23)
Supplement: Supplementary file 6 [file PLD3-1-e00023-s006.pdf]

Genotype	Taxa	Photoperiod	Type
PI 329311	PI 329311	Sensitive	Energy
PI 213900	PI 213900	Insensitive	Energy
PI 505735	PI 505735	Insensitive	Sweet
PI 329632	PI 329632	Sensitive	Energy
PI 35038	PI 35038	Insensitive	Energy
PI 585954	PI 585954	Sensitive	Energy
NTJ2	none	Sensitive	Energy
M81e	PI 653411	Sensitive	Energy
PI 229841	PI 229841	Insensitive	Energy
PI 297155	PI 297155	Insensitive	Energy
PI 506069	PI 506069	Sensitive	Energy
PI 508366	PI 508366	Sensitive	Energy
PI 297130	PI 297130	Sensitive	Sweet
Grassl	PI 154844	Sensitive	Sweet
PI 152730	PI 152730	Sensitive	Energy
PI 195754	PI 195754	Insensitive	Sweet
BTx623	PI 564163	Insensitive	Grain
CK60B	none	Sensitive	Grain
B.Az9504	PI 656003	Sensitive	Sweet
San Chi San	PI 542718	Insensitive	Sweet
ICSV700	none	Sensitive	Sweet
Atlas	PI 641807	Insensitive	Sweet
Leoti	PI 641825	Insensitive	Sweet
Chinese Amber	PI 22913	Insensitive	Sweet
Della	PI 566819	Insensitive	Sweet
Rio	PI 651496	Insensitive	Sweet
PI 642998	PI 642998	Insensitive	Energy
China 17	none	Insensitive	Sweet
PI 510757	PI 510757	Sensitive	Energy
PI 655972	PI 655972	Insensitive	Energy

Origin	Race
Ethiopia	Durra
Kenya	Bicolor
Zambia	Caudatum
Ethiopia	Durra
United States	Caudatum
Togo	Guinea
India	Durra
United States	Caudatum/durra
South Africa	Kafir
Uganda	Kafir
Togo	Guinea/bicolor
Mali	Guinea
Uganda	Caudatum
Uganda	Caudatum
Kenya	Caudatum/bicolor
China	Kafir-bicolor
United States	Kafir
United States	Kafir
United States	Kafir
China	Bicolor
NA	Bicolor
United States	Durra
United States	Durra
China	Caudatum/durra
United States	NA
United States	Durra
NA	Bicolor
China	Bicolor
Cameroon	Durra
South Africa	kafir
