# Supplementary figures and images for: High‐throughput profiling and analysis of plant responses over time to abiotic stress
Source: Plant Direct. 2017 Oct 25;1(4):e00023. doi: 10.1002/pld3.23 (PMC6508565; doi:10.1002/pld3.23)

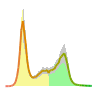

Supplement: Supplementary file 9 [file PLD3-1-e00023-s009.gif]
